# Comparative Structural Analysis of GFRP, Reinforced Concrete, and Steel Frames under Seismic Loads

**DOI:** 10.3390/ma16144908

**Published:** 2023-07-09

**Authors:** Luca Mincigrucci, Marco Civera, Erica Lenticchia, Rosario Ceravolo, Michele Rosano, Salvatore Russo

**Affiliations:** 1 Department of Structural, Geotechnical and Building Engineering, Politecnico di Torino, Corso Duca degli Abruzzi 24, 10129 Turin, Italymarco.civera@polito.it (M.C.); erica.lenticchia@polito.it (E.L.); rosario.ceravolo@polito.it (R.C.); 2 School of Civil and Mechanical Engineering, Curtin University, Kent St., Bentley, WA 6102, Australia; m.rosano@curtin.edu.au; 3 Department of Architecture Construction Conservation, Università IUAV di Venezia, Santa Croce 191, 30135 Venezia, Italy

**Keywords:** pultruded GFRP element, seismic design, GFRP structural performance, storey drift, GFRP frame

## Abstract

Fibre-reinforced polymer composites in general, and especially glass fibre-reinforced polymer (GFRP), have increasingly been used in recent decades in construction. The advantages of GFRP as an alternative construction material are its high strength-to-weight ratio, corrosive resistance, high durability, and ease of installation. The main purpose of this study is to evaluate the response of GFRP under dynamic conditions (more specifically, under seismic loads) and to compare the performance of this composite material with that of two traditional building materials: reinforced concrete and structural steel. To this aim, a finite element analysis is carried out on a two-dimensional frame modelled with steel, reinforced concrete (RC), or GFRP pultruded materials and subjected to a seismic input. The dynamic response of the structure is evaluated for the three building materials in terms of displacements, inter-storey drift, base shear, and stress. The results show a good performance of the GFRP frame, with stress distribution and displacements halfway between those of RC and steel. Most importantly, the GFRP frame outperforms the other materials in terms of reduced weight and, thus, base shear (−40% compared to steel and −88.5% compared to RC).

## 1. Introduction and State of the Art

The use of glass fibre-reinforced polymer (GFRP) has been developed over the past decades for different applications, including bridges and building construction [1]. The employment of this innovative material to strengthen existing structures is also becoming more and more common, especially for improving seismic performance [2,3]. The advantages of FRP as an alternative construction material are its high strength-to-weight ratio, corrosive resistance, high durability, and ease of installation. Its relatively low weight, when compared to steel and, especially, reinforced concrete (RC), reduces the total weight of the structure, with important consequences from a structural perspective, particularly in the design of buildings with limited foundations. As will be proved in this article, this reduces the resulting base shear force, an aspect that represents a major beneficial factor to improve the seismic design of a structure. On the other hand, the pultrusion process may cause manufacturing imperfections; however, these can be detected, localised, and assessed, similarly to any other material defect [4,5].

This research aims to compare the results of a dynamic structural analysis of a GFRP frame with similar structures made of more traditional materials: RC and steel. More specifically, the focus of this research work is on seismic loads. In fact, the seismic performance of GFRP structures has only fairly recently been researched.

While the mechanical response of this composite material under static loads is relatively well known, its dynamic characterisation is not widely understood or published. However, some studies on the dynamic parameters of composites have been carried out; mainly, Russo and Boscato [6] performed experimental tests in the free vibration field on simply supported pultruded beams subjected to flexural, transversal, and torsional vibrations. Some values for the damping ratios for different types of cross-section beams were proposed. A large pultruded structure was built in L’Aquila (Italy) to temporarily protect the church of Santa Maria Paganica which was damaged during the 2009 earthquake [7]. This structure is made entirely of pultruded GFRP elements. As a consequence, the current codes and guidelines for the design of FRP structures and FRP-reinforced structures (including, but not limited to GFPR ones) do not have a comprehensive seismic section, either in the USA, Canada [8], European Union [9], or other countries.

For what specifically concerns the earthquake resistance of GFRP-made structures, other very recent works include Ghomi and El-Salakawy’s studies on the seismic behaviour of exterior GFRP-RC beam–column connections [10], GFRP-RC interior beam–column–slab subassemblies [11], and GFRP-RC moment-resisting frames [8]. Also worth mentioning is Tavassoli and Sheikh’s work on the seismic resistance of steel-GFRP reinforced circular columns [12]. However, different from this research work, all these contributions focused on the combined use of GFRP with RC, with GFRP acting as a strengthening system. Here, instead, the use of a fully GFRP multi-span, multi-bay frame is investigated in total replacement of the more conventional building materials for new constructions.

The rationale for the use of GFRP in earthquake engineering is that, even though the material exhibits anelastic-brittle behaviour, GFRP-made structures show a progressive reduction in displacement, highlighting a certain dissipative mechanism and allowing a significant reduction in the design requirements necessary for resisting seismic force. Hence, GFRP frames have several mechanical and dynamic properties that are suitable for seismic design. In general terms, for many commonly used structural configurations, the values of natural frequency and modal damping indicate good structural behaviour, even when compared to traditional materials such as steel, aluminium, wood, or concrete. In addition, their low weight and long period of vibration are significant benefits for the application of GFRP structures in seismic engineering applications.

Despite the absence of ductility and dissipation capacity in pultruded frames, using GFRP profiles provides other benefits for structural design in seismic areas. The reduction in weight of the whole structure makes it possible to use smaller and/or shallow foundations. Furthermore, in comparison with a steel frame, GFRP structures show a similar performance in terms of global deformability, but with reduced shear forces due to the lower linear mass density of the frame.

## 2. Settings of the Finite Element Models and Analyses

As mentioned, numerical simulations are carried out on three equivalent, two-dimensional frames using ABAQUS 6.12. The target frame is modelled in GFRP, while the other two FE models, intended as benchmarks, are representative of steel and RC structures. The aim is to make direct comparisons between these three construction materials.

All the performed analyses of the three materials consider elastic–linear constitutive relations. Due to the magnitude of the accelerations expected during major earthquakes, multi-span, multi-bay structures are likely to experience non-linear plastic deformations (especially RC frames). This notwithstanding, comparing the frames’ linear elastic responses is a necessary preliminary study, required before any further complex non-linear analysis is carried out, especially in the absence of more detailed experimental knowledge of the plastic behaviour of GFRP frames.

In this work, two analyses are performed:

1. As a first and preliminary step, a modal analysis is performed for all frames and comparing the fundamental modes of vibration in terms of natural frequencies and mode shapes.
2. Then, a dynamic analysis is carried out, applying the time history of a horizontal component of a recorded acceleration from the 1995 Kobe earthquake.

This latter analysis is discussed in more detail in the following subsection.

### Comparative Dynamic Analysis

Under the seismic conditions of the Kobe earthquake (1995), the displacements and inter-storey drift ratio are evaluated to assess the overall dynamic response of the frames. In addition, the base shear and Von Mises equivalent stress are compared for the three construction materials to provide an assessment of the structural performance of all frame designs. The dynamic characterisation of all three materials is then discussed, and frame design is explained, considering the equivalent performance criterion. The acceleration data, summarised in Table 1, were retrieved from the PEER Ground Motion Database [13] under the code name KOBE-JMA

After defining the amplitude of Kobe acceleration in the time domain, ABAQUS applies the earthquake to the model as acceleration boundary conditions in the horizontal plane. Dynamic analysis is then performed using the implicit method. In this study, dynamic analyses are performed under the hypothesis of large displacements. Actually, the geometric non-linearity could be considered negligible for the frame being studied but an analysis under the large displacement hypothesis is usually more accurate; therefore, the current nodal positions in the current configuration are used to define the element formulation. A full Newton technique is used to solve the non-linear dynamic problem.

## 3. Properties of the GFRP Structural Components

**Figure 1:**
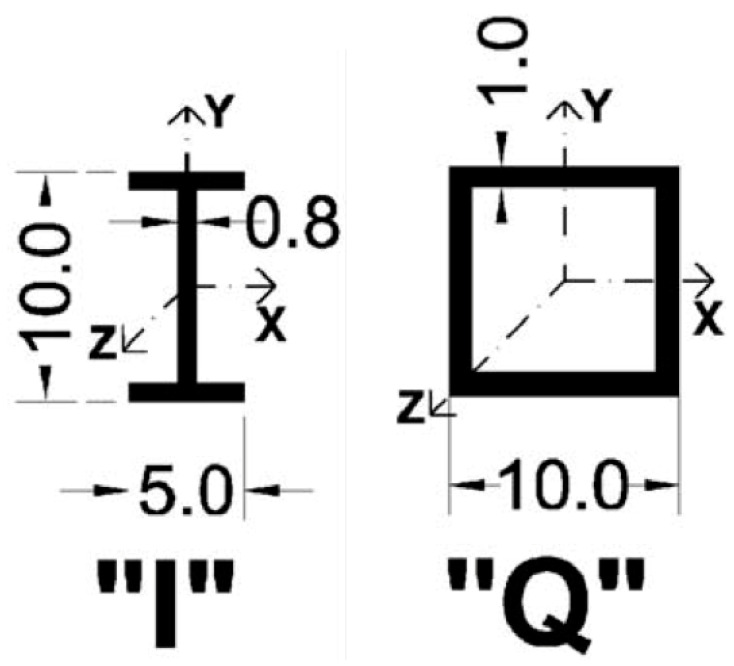
The two cross-sections considered for the GFRP frame.

To evaluate the dynamic behaviour of an all-GFRP frame, it is first necessary to discuss the properties of its components. In particular, it is important to have some general indications of the dissipation capabilities of such structural elements. In fact, whilst the damping values for RC and steel are widely known, little research on GFRP damping is available in the scientific literature. Russo and Boscato [6] performed experimental tests on the free vibrations of pultruded GFRP elements, proposing damping values for different cross-section beams. In this work, the same fibre/matrix ratio (40–60%) is assumed. The experimental results reported by them for two different cross-section beams are numerically replicated to provide damping values for the next step of the numerical simulation. Hence, two cross-sections investigated in [6] are chosen for the analysis: the I-beam (considering the direction of the strongest moment of inertia $J_{max}$) and the rectangular boxed section Q, depicted in Figure 1. The characteristics of the cross sections are summarised in Table 2. Concerning the material longitudinal tensile strength, Russo and Boscato [6] do not report an exact value but a range between 200 MPa and 500 MPa. As noticed in Russo and Boscato [6], Ghomi and El-Salakawy [11], and many other experimental studies, GFRP maintains a linear elastic behaviour until failure, with negligible or no plastic deformation. Hence, it is feasible to numerically model it with a purely linear elastic constitutive law [10].

The analysed structural profiles are modelled in ABAQUS 6.12 using the B23 beam element, which uses the Euler–Bernoulli theory and cubic interpolation. Boundary conditions are set as simply supported, according to the conditions of the experiment. Mechanical properties defining the anisotropic and transversally isotropic behaviour of GFRP are reported in Table 3. These experimental values are retrieved from [6].

### 3.1. Damping of Pultruded GFRP Beams

To be properly inserted in ABAQUS, the viscous damping of the GFRP beams needs to be defined as Rayleigh damping, characterised by the conventional mass- and stiffness-proportional α and β coefficients. These parameters can be defined from the damping ratio ξ, experimentally known for this structural element. Using the damping ratios 2.59% and 2.720% proposed for I-beam and Q-beam, respectively, and their first two natural pulsations, $\omega_{1}$ and $\omega_{2}$, the α and β coefficients can be calculated as reported in Table 4. As before, these values are based on experimental findings retrieved from [6] and [14] on similar applications using GFRP beams of identical manufacturing procedures and pultrusion.

To evaluate the reliability of the so-obtained GFRP simply supported beam models, an impulsive load is imposed on the middle of the beam to simulate impact force. Then, the time history of the acceleration of the middle point is evaluated, representing the free vibration response. Since the simulated free vibrations match well those recorded during the experiments, the approach is considered verified, and the damping values proposed by Russo and Boscato [6] are considered reliable for the numerical analysis. Thus, these values will be considered in the next step of modelling to define the all-GFRP frame damping properties, with adjustments that will be discussed later on.

## 4. Design of the Frames

**Figure 2:**
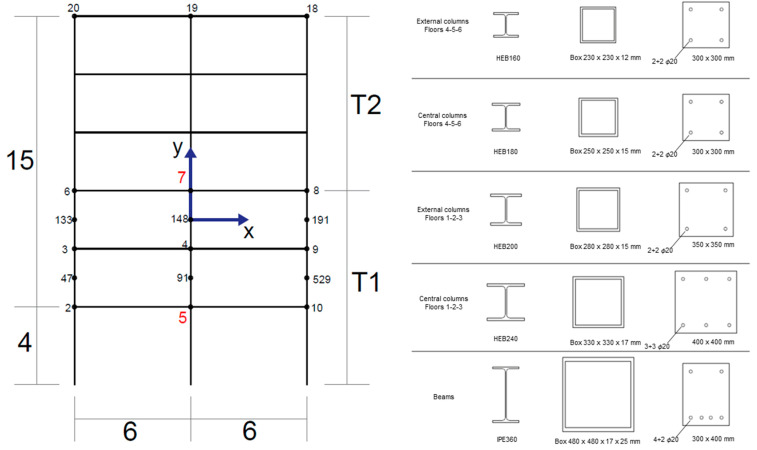
The frame geometry (equal for all materials) with the monitored points’ positions and the cross sections for the steel, GFRP, and RC beams and columns.

The geometry of the 2D, six-storey, two-bay frame illustrated in Figure 2 is considered the target structure for finite element analyses. This is assumed to be identical to the frame proposed by Diaferio and Foti [15] who analysed a two-dimensional steel frame subjected to several impulsive motions, including the Kobe earthquake used for this study.

All the restraints between the structural elements are under clamped conditions. As will be better detailed later on, the three frames are designed to provide an equivalent structural performance to static loads, allowing for a fair and reliable comparison between the three materials.

To fully replicate the structure and load distribution reported in Diaferio and Foti [15], two contributions were considered for dead loads: the frame self-weight (permanent structural loads) and an arbitrary value of 20,000 N, distributed uniformly along the length of each horizontal beam to simulate permanent non-structural loads.

### 4.1. Steel Frame

As stated above, the steel frame is intended to replicate the study proposed by Diaferio and Foti [15]. This applies to the single steel profiles composing the steel frame as well. These are summarised in Table 5, considering the IPE profiles for all beams and HEB profiles for all columns, with different dimensions. The mechanical properties assumed for the structural steel are reported in Table 6, alongside the ones of the RC frame, which will be discussed in the following subsection. The frame is expected to behave linearly; nevertheless, a common s235 structural steel, with a yield stress f_yk_ = 235 MPa, was assumed.

### 4.2. Reinforced Concrete (RC) Frame

The mechanical characteristics of concrete have already been reported in Table 6, considering a typical concrete subclass of C25/30. According to Eurocode 2 and EN 206-1, that concrete grade refers to f_ck_ = 25 MPa compressive strength based on the cylinder test and f_ck,cube_ = 30 MPa compressive strength based on the cube test. A standard B450C steel was assumed for the reinforcement bars with a yield stress f_yk_ = 450 MPa. Damage-induced stiffness reduction and non-linear (post-yielding) deformations are not included in the model. These approximations err on the side of caution, as they underestimate the total displacements of the RC frame, thus making the benchmark for the GFPR frame even stricter and more compelling.

For the RC frame, all elements—both horizontal beams and vertical columns—are designed considering the equivalent structural performance of the steel frame.

Since the performance of the horizontal beams is primarily governed by the bending moment, an equivalent rigidity approach is followed. That is to say, the equivalent beam cross-section is obtained by comparing the flexural stiffness of the steel beams with the flexural stiffness of a homogenised concrete cross-section. In this way, the minimum required moment of inertia J of the concrete beam can be found. The moment of inertia of the section homogenised to concrete is hence calculated under pure bending conditions. Unfortunately, this does not guarantee equivalent cross-section strength under other kinds of solicitation. The corresponding chosen RC cross-section is rectangular with a size of 30×40 cm and with minimum reinforcement. This minimum amount of reinforcement is provided for all the sections, according to the current European norms [16].

RC columns are designed considering a simplified flexural rigidity EJ, designed for buckling load as reported in Equation (1), where $E_{cd}=E/1.2$, $J_{c}$ is the moment of inertia of the full concrete cross-section, and φ is the non-dimensional creep coefficient of concrete, chosen according to the diagram in Figure 3.1 of Eurocode 2 [16].

(1) $$EI=\frac{0.3}{1+0.5\varphi }E_{cd}J_{c}$$

Table 7 and Table 8 report the beam and column cross-sections with respective reinforcement (reported in terms of the diameter of the steel bars ϕ, in mm). The geometrical equivalent cross-sections for every element of the frame are illustrated on the right side of the previously reported Figure 2. Square cross-sections are considered for the columns, while the aforementioned 30×40 cm rectangular cross-section is used for all beams.

#### Equivalent Full-Concrete Cross-Sections

For the RC frame, the finite element model of interest is a two-dimensional model with composite cross-sections (concrete plus reinforcement steel bars). However, the RC frame was modelled in the ABAQUS environment with homogenised full-concrete cross-sections that consider the presence of steel reinforcement. In more detail, the equivalent RC beam is obtained by equalling the moment of inertia of the homogenised cross-section, reported in Table 7, to the moment of inertia of a full section of concrete, providing the same response to the bending moment. Similarly, since the frame columns are mainly subjected to compressive loads, an equivalent area of concrete is considered, defining a homogenised cross-section that takes into consideration the reinforcement steel contribution to the resistance to axial loads. All the equivalent cross-sections composing the frame in the finite element model are summarised in Table 9.

### 4.3. GFRP Frame

The mechanical characteristics of the chosen composite material have already been reported in Table 3. Equivalent structural performance criteria are found for every structural element in the frame, both for vertical columns and horizontal beams.

In detail, GFRP beam cross-sections are designed to have the same displacement as that of steel beams; therefore, the two maximum elastic transverse displacements are set to be equal and the minimum required moment of inertia J of the GFRP beams can be found.

As before, the buckling load is the most restrictive parameter to consider when assessing the behaviour of columns subjected to compressive load, especially for slender beams with open, thin-walled profiles which are common for structural steel and GFRP [17]. To determine the optimised cross-section of the GFRP columns, the Euler critical load for steel columns is set equal to the equivalent Euler formula for GFRP profiles, as reported by Equation (2), where $L_{0}$ is the column’s effective length and considering the specific restraint conditions (fixed at both ends). From the equivalence, the minimum required moment of inertia J of GFRP columns can be found.

(2) $$\frac{\pi^{2}\;E_{steel\;}J_{min,\;steel}}{L_{0,steel}^{2}}=\frac{\pi^{2}\;E_{GFRP}\;J_{min,GFRP}}{L_{0,GFRP}^{2}}$$

GFRP box sections are chosen for both columns and beams, satisfying the minimum moment of inertia required to provide equivalent structural performance. Similarly, to the RC frame, square box sections of different sizes are chosen for columns, while a unique rectangular box section is applied to all beams. All cross-sections’ dimensions are portrayed in Figure 2 and summarised in Table 10, where h is the height, b is the base width, $t_{w}$ is the web thickness, and $t_{f}$ is the flange thickness of the steel profiles.

### 4.4. Damping Evaluation of the Entire Frames

Previously, the estimation of the viscous damping was discussed for GFRP simply supported beams, aiming at reproducing the experimental findings of Russo and Boscato [6]. However, these need to be corrected for different boundary conditions; furthermore, similar values must be defined for the steel and RC frame as well.

As seen before for GFRP, it is assumed that, for a given material, the damping ratios related to the first two vibration modes are equal. With this assumption, the equivalence $\xi_{1}=\xi_{2}=\xi$ can be considered not only for GFRP but also for RC and steel. The damping ratios assumed in this simulation for each material are reported in Table 11.

For the RC frame, a damping ratio equal to 5% is used. This value is typical for reinforced concrete structures in which the presence of cracks is assumed. For the steel frame, a value of 2% is considered. This is generally associated with welded steel structures. The issue is more complex regarding the frame in GFRP. The value of ξ for box sections reported in Section 3.1 is related to experiments conducted on a simply supported beam, while all the restraints between the beams and columns that form the frame in GFRP and between the columns and ground are assumed as perfectly clamped. This difference in constraint can result in different values of the damping ratio and, consequently, in the outcomes of subsequent analyses. Moreover, the damping ratios considered for RC and steel are defined assuming the presence of defects in these materials, while for GFRP the effects of such potential material imperfections are not directly accounted for. In the absence of experimental data related to GFRP clamped–clamped beams, a parametric analysis was performed on the damping ratio: the ξ relative to the simply supported beam is considered alongside with the same values increased and reduced by ±2.5%—corresponding to (c) and (e) in Table 5. Since the α and β coefficients depend on the critical damping ratio and pulsations of the first two vibration modes, Rayleigh damping is evaluated for each structural element on the whole frame. Every member of the frame has its own length and different cross-section; therefore, it has different damping coefficients. Each beam and column are considered perfectly clamped, and the first two natural frequencies are calculated with modal analysis to estimate the α and β values. This procedure is carried out for the entire frame.

## 5. FE Analysis Results and Discussion

The results of the numerical simulations for the different cases described before are here reported and discussed.

### 5.1. Preliminary Test: Free Vibrations of Pultruded GFRP Elements

As a preliminary test, the experimental results retrieved from [6] are compared to the FE analysis described in Section 3.1, considering the free vibrations of pultruded profiles after the application of a simulated impact load, in terms of normalised acceleration.

**Figure 3:**
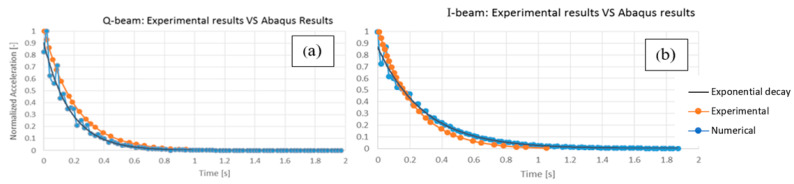
Comparison between the numerical analysis (light blue line) and experimental results (orange) for (**a**) the Q-beam and (**b**) the I-beam. The black line represents the exponential decay law that best fits the numerical results.

The Q-beam and I-beam experimental results reported by Russo and Boscato [6] are reported in Figure 3, alongside the FE analysis results. These highlight the strong similarities between the experimental data and the numerical simulations. Figure 3b shows the I-beam’s free vibration response. The interpolation line of the acceleration peaks decreases with exponential law as time increases due to the damping effect of the material. The acceleration values before 0.1 s are disregarded because of the impact load interference in the first part of the simulation which causes a disturbance to the acceleration recording. As can be seen, the two curves match quite well, with a maximum difference of 15.5% (due to the aforementioned reasons) considering the whole time series. The maximum difference is reduced to 5.3% if the part before 0.1 s is omitted, as said, with this maximum divergence happening in the central part of the curves at 0.43 s. The overall response of the analysed I-beam under free vibration can be considered practically the same for both the numerical simulation and experimental analysis. A comparison between the numerical analysis and experimental results for the box Q-beam is shown in Figure 3a. Again, the maximum value occurs at the initial instant (t = 0.00 s) and then the acceleration fades away according to an exponential law. The two curves show a similar behaviour at any time, with a maximum difference of 12.1% at 0.06 s.

For all these reasons, the numerical simulation of the GFRP beams’ free vibrations is considered satisfactory. The discrepancies between the numerical approach and the experiments are considered negligible. In particular, the value of 2.720% corresponding to the Q-beam is used for the next step of the analysis, evaluating the overall damping effect of the all-GFRP frame. In addition, as mentioned previously, deviations of ±2.5% of this value have been considered to investigate the sensitivity to different conditions between the experiment of Russo and Boscato [6] and the frame under study.

### 5.2. Modal Analysis of Pultruded GFRP Elements

The results of the modal analysis are shown in Table 12. The natural frequencies of the first five vibration modes and their corresponding periods are extracted for all five frames. As can be seen in Table 12, all frames show a relatively short natural period of vibration, less than 1 s. The steel frame has the shortest natural period (0.34 s), while the RC provides the largest one (0.52 s). The natural modes of the GFRP frames with the different damping ratios, (c), (d), and (e), are practically superimposed. In fact, it is proved that in the Rayleigh formulation, by varying the damping ratio, the mode shapes remain unchanged and the frequencies experience minimal variations, which are negligible for the purposes of this analysis. The steel frame is characterised by relatively high frequencies, with the risk of resonance with high-frequency earthquakes, while the GFRP and RC have lower frequency values. As a first result it can be said that, in terms of natural vibrations, the GFRP frames seem to have an intermediate behaviour between steel and RC, with frequencies and periods of vibration more similar to RC than steel. For what concerns the mode shapes, these have a similar configuration for the steel, RC, and GFRP frames.

### 5.3. Time-History Analysis—Base Shear

**Figure 4:**
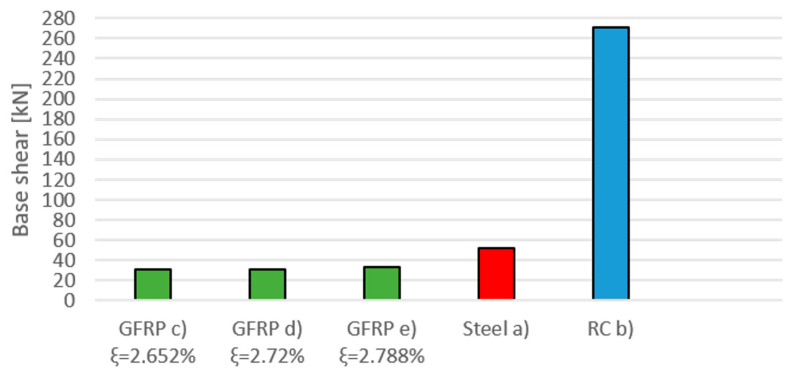
Maximum base shear for all frames.

A time-history analysis is performed under the hypothesis of large displacements. Base shear is evaluated as the horizontal reaction force at the bottom of the three ground-floor columns. The maximum base shear values for all frames are reported in Table 13 and plotted in Figure 4. As one can see, the value is much higher for RC. This is understandable as the base shear is mostly dependent on the mass of the structure. In this case, the total weight of the frame was 6046.36 kg for the GFRP one; 7396.20 kg for the steel one; and 39,390.75 kg for the RC one. It is evident that the latter frame has a considerably higher mass and subsequently higher base shear, while the GFRP is the most lightweight option; the minimum value, which corresponds to the lowest damping ratio ξ = 2.652%, is highlighted in bold.

The maximum base shear occurs in the central column for all frames. The GFRP frame characterised by the lowest damping ratio shows the lowest max base shear amongst all the other frames, followed by the other GFRP frames with a short gap. The steel frame corresponds to an intermediate value, while the RC frame is affected by the highest value. This trend reflects the weight of the structure, which is heavier for RC and lighter for the composite. The higher mass of the RC frame results in a higher base shear by comparison with steel and GFRP, whose structures are 20% and 15% of the total weight of reinforced concrete, respectively. Considering the lowest value of the maximum base shear in GFRP frames, this is approximately 59.5% and 11.5% of the corresponding values for steel and RC structures. The lower linear mass density of composites enables a reduction in the shear force at the ground floor (i.e., at the columns’ base level), with important positive consequences for structural design, such as size reduction and reductions in the required foundations.

### 5.4. Time-History Analysis—Inter-Storey Drift

In a two-dimensional model, the structural response of RC, GFRP, and steel frames subjected to ground motions can be assessed through displacement. The inter-storey drift $\delta_{max}$ is evaluated and compared for all five frames, defined as the maximum relative translational displacement between two consecutive floors [19]. The inter-storey drift can also be expressed as a percentage of the storey height, as suggested by Diaferio and Foti [15].

**Figure 5:**
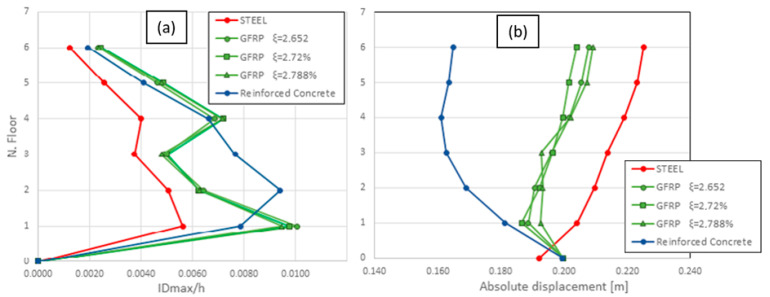
Inter-storey drift ratio (**a**) and absolute displacements (**b**) for all frames.

Figure 5a shows the inter-storey drift ratio for each frame in large displacement hypotheses. The maximum inter-storey drift ratio occurs between the first and the ground floor for steel and GFRP frames, while RC shows a bigger value between the second and first floors. The massive nature of the RC frame makes the structure behave like a rigid body, especially on the lower floors where the inter-storey drift ratio range is between 0.007 and 0.010. The GFRP (c) frame shows the maximum inter-storey drift ratio, which is 0.0101, corresponding to the first floor. The overall trend of the analysis shows that all the frames have nearly the same values at the top floor, with an inter-storey drift ratio of 0.002, and higher values at lower floors, excluding only the RC, which has the maximum inter-storey drift ratio at the second floor.

The seismic structural response can also be assessed in terms of absolute displacement, which is the horizontal displacement of a specific node from its original equilibrium position. The evaluation of the maximum absolute displacement for each floor provides a general idea of the overall structural deformability. The highest value of lateral displacement during the earthquake is exhibited by the steel frame: as shown in Figure 5b, the maximum value of 0.225 m occurs in correspondence with the top floor, and displacements decrease at lower floors. The RC frame exhibits an opposite trend, with maximum values at the bottom, except for the last two floors that follow the steel trend. The GFRP frames, which show almost coincident trends, have a more uniform displacement distribution along the structure’s height, containing displacements between 0.18 and 0.21 m. In general, it can be observed that the GFRP frames, as far as absolute displacements are concerned, have an intermediate behaviour between the two more traditional materials with a general trend more similar to steel than to RC.

### 5.5. Time-History Analysis—Stresses

The time histories of Von Mises equivalent stress are calculated for all five structures in a time-history analysis, following the conventional formulation recalled in Equation (3):

(3) $$q=\sqrt{\frac{3}{2}S_{ij}S_{ij}}$$

where $S_{ij}=\sigma_{ij}+p\delta_{ij}$, $p={-\frac{1}{3}\sigma }_{ii}$ and $\delta_{ij}$ is the Kronecker delta.

**Figure 6:**
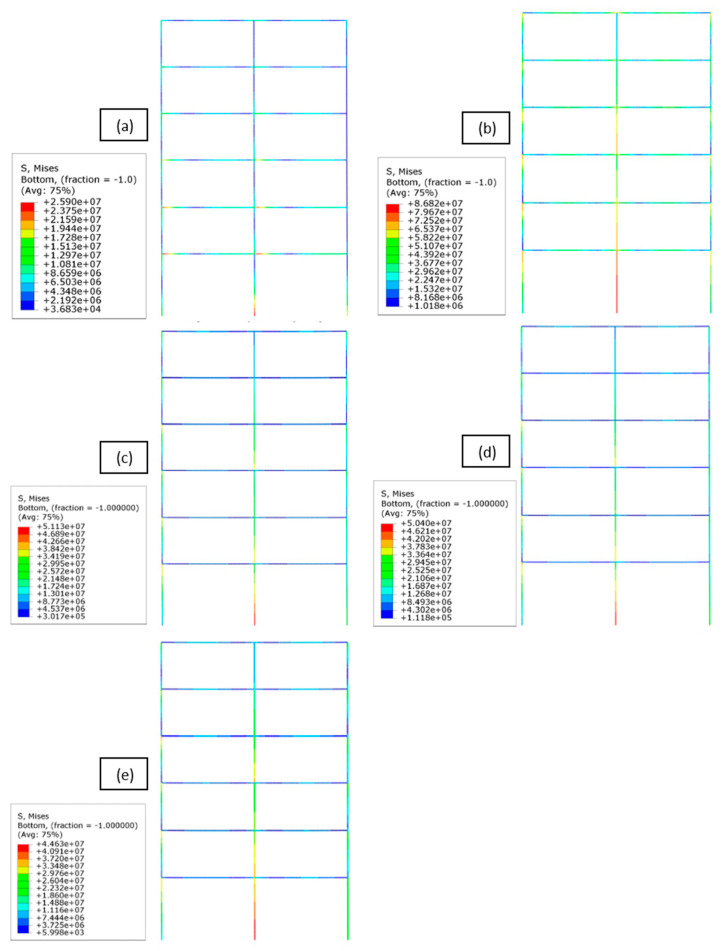
Stress contours at 15 s for the different frames: reinforced concrete (**a**), steel (**b**), GFRP with ξ = 2.652% (**c**), GFRP with ξ = 2.720% (**d**), and GFRP ξ = 2.788% (**e**).

The contour graphs allow for a general understanding of the stress distribution in the entire frame. Time is the fixed parameter, and the stress level is shown in every element of the frames at 15 s. Since maximum accelerations of the Kobe earthquake occurred between 5 and 15 s, these moments are representative of the time-history analysis. In Figure 6, the five frames are compared at the same time, showing differences in stress distribution and intensity. The most stressed point of all the frames is the bottom point of the ground floor central column and, in particular, among the five frames, the steel one is characterised by higher stress values. Stresses in the RC frame are smaller, and the most stressed point is the beam–column connection on the first floor, reaching a value of 26 MPa. Despite the differences in terms of magnitude, while RC and steel show a homogeneous distribution among the frame’s elements, GFRP provides higher values along the columns and beam–column joints at every floor, and lower stresses along the beams.

In total, 18 critical points are chosen to observe the stress variation through time. Each point position in the frame is as previously illustrated in Figure 2. Since higher stresses occur in the lower part of the frame, the chosen points are beam–column joints at the first (nodes 2, 5, and 10), second (nodes 3, 4, and 9), and third floor (nodes 6, 7, and 8). Then, mid-floor nodes between the first and second floors, and between the second and third floors, are selected (nodes 47, 91, and 529 and 133, 148, and 191). To have a clear idea of the entire frame, beam–column joints at the top floor are also considered (in the same order, nodes 20, 19, and 18). For brevity’s sake, we only report the nodes characterised by the most significant stress of the whole frame, namely nodes 5 and 7.

The general trend of the time-history analysis for the Von Mises equivalent stress points out that peak values are reached at around 8 s and 15 s, corresponding to the time in which the maximum accelerations of the Kobe earthquake are recorded. All three materials show similar overall behaviour, where maximum stresses occur at the beam–column joints in the lower floors and decrease with increasing height. From the graphs of the stresses in nodes 5 and 7, it emerges that the three frames in GFRP have a very similar trend and are basically the intermediates between the steel and RC frames.

The central column on each floor is the most critical element. As reported in Figure 6a, the RC frame reaches its maximum stress value of 52.72 MPa at node 5 (corresponding to the central column–beam joints of the first floor, see Figure 2) which is 5–10% bigger than the external ones. The stress in the central column decreases on the second and third floor, which is 47.81 MPa and 39.27 MPa, respectively, at nodes 4 and 7, reaching the minimum value of 17.53 MPa on the top floor (node 19). In the mid-floor points, the stress is less than 80% compared to the correspondent nodes on the lower and upper floors. Even if the average stress value has a short range of variation and is lower than the GFRP frame, RC exhibits higher peaks during maximum earthquake accelerations, drastically exceeding the average value, especially at lower floors. In the steel frame, the same node 5 records the maximum Von Mises equivalent stress at 151.87 MPa, below the assumed yielding point. This is also verified throughout the rest of the frame, with again the mechanical stress decreasing in the central column at the second, third, and last floor (127.93 MPa at node 4, 128.04 MPa at node 7, and 106.15 MPa at node 19).

**Figure 7:**
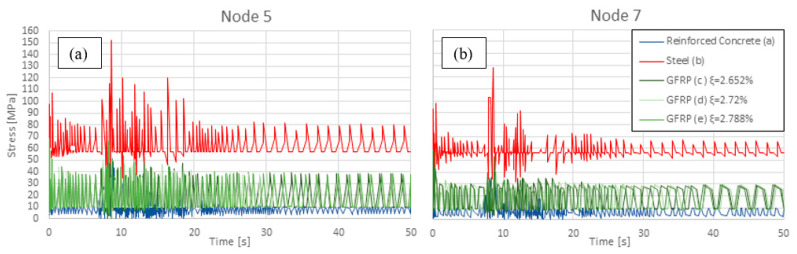
Stress time history in nodes 5 (**a**) and 7 (**b**).

Node 5 is the one subjected to the highest stress in the GFRP frames as well (67.77 MPa for ξ = 2.652%). This value does not decrease considerably on the upper floors, remaining between 50 MPa and 60 MPa until the third floor and then strongly decreasing until a minimum of less than 20 MPa is reached at the top floor. All three GFRP frames are characterised by a homogeneous stress distribution over the entire earthquake duration, with average values between 10 MPa and 40 MPa for node 5, Figure 7a, and between 10 MPa and 30 MPa for node 7, Figure 7b, without significant peaks. Therefore, the whole frame is verified at any node and at all times, even considering the lowest point in the range of the material longitudinal tensile strength (200 MPa).

The GFRP frames seem to be less sensitive to the acceleration variation due to the ground motions, even during the interval characterised by the highest values of acceleration, between 8 s and 15 s. Steel frame stresses are the highest compared to RC and composite. Each node of each floor shows a maximum stress value higher than 100 MPa, with peaks of 152 MPa in node 5. The Von Mises equivalent stress in the steel elements is almost double that in the RC and GFRP ones. GFRP and RC show comparable results in terms of stresses for the entire frame, though the RC frame exhibits 20–30% lower values for all the floors. However, the steel frame exhibits stress at least twice as high compared to those of the RC and GFRP ones. The stress time-history analysis of the five frames highlights the different stress distributions in significant nodes of the structures, in particular, beam–column joints, which were characterised by the highest stresses, especially those on the lowest floors. Although the RC and GFRP frames show similar average behaviour in terms of stress trends, the steel frame exhibits significantly higher values at specific instants, i.e., when the intensity of the earthquake is stronger.

## 6. Discussion

This study compares the dynamic behaviour of five two-dimensional frames, investigated in their linear elastic field and under the hypothesis of large displacements. An initial comparative study was performed between traditional materials (structural steel and reinforced concrete) and GFRP. Furthermore, concerning the GFRP frame, three values of damping values were considered and evaluated through a parametric analysis.

In analysing the first comparison, it is possible to make the following comments. The natural periods of the three frames are all included in the 0–1 s range. The steel frame presents higher natural frequencies, followed by the RC and the GFRP, with the only exception of the first mode.

Concerning the base shear, the composite material is undoubtedly the one with the best performance. The reason is certainly to be found in the lower mass density and thus the lower weight of the whole frame, as already mentioned in earlier sections. Figure 4 clearly shows the gap between the mechanical stress that affects the GFRP frames and the one that affects the reinforced concrete frame, which is almost nine times greater.

Another crucial quantity to be analysed when studying the dynamics response of framed structures and buildings is the inter-storey drift. In Figure 5a, the inter-storey drift ratios of the three materials are compared. The diagram shows that the composite material has the largest inter-storey displacement at the first and the last three floors. This is certainly a negative aspect, as high inter-storey displacements are typically associated with damage in the infill walls and non-structural components. In general, the RC is affected by the smallest displacements. On the other hand, Figure 5b evidences the overall structural deformability. In this case, the composite material shows a behaviour that is halfway between the two traditional materials, with a deformation similar to that of the metal frame.

Finally, regarding the Von Mises equivalent stress on the frames, the focus of this analysis was at 15 s of the time history; as said, this corresponds to a maximum of the input acceleration for the given seismic time history. Figure 6 shows how the frames have a peak in stress in the same position but with different intensities. Moreover, having selected the critical points for analysis, the stress time histories for the different materials were plotted. Steel is the material undergoing the largest amount of mechanical stress, followed by GFRP and RC. The composite material, despite higher stress values compared to RC, has the advantage of a more regular time history, without the peaks that afflict the other materials.

The second comparison refers to frames made of the same GFRP material. From the parametric analysis performed on the damping ratio, the following additional comments can be made. First and foremost, from the comparison in terms of the maximum base shear (Figure 4), it emerges that frame (c), characterised by the lowest value of damping ratio, is the least stressed frame, also with respect to the other materials.

In terms of displacements, the three frames in GFRP are characterised by very similar trends and values.

Similarly, the small differences in damping do not cause significant variations in the time history of Von Mises equivalent stress, as can be seen from the response at nodes 5 and 7 (Figure 7).

## 7. Conclusions

The numerical study reported here represents, to the best of the authors’ knowledge, the first structural analysis and direct comparison of GFRP, reinforced concrete, and steel multi-storey, multi-bay frames. This makes it a useful tool to correlate these more traditional construction materials and GFRP. It also represents the first step for the evaluation of the real seismic responses of such frames.

In light of the results obtained from this numerical analysis, the following conclusions can be drawn:

- The choice of composite material for the frame reduced the base shear by 40% compared to steel and 88.5% compared to RC, a natural consequence of the lower mass density of GFRP compared to its competitors. On the one hand, this outcome confirms what is qualitatively expected from current state-of-the-art materials. On the other hand, this direct comparison quantitatively assesses the very large benefits of GFRP over steel and, especially, RC. This leads to important positive consequences in terms of structural design under seismic conditions, i.e., a reduction in the total size of foundations required, with significant savings in the design and construction phases. This can be seen as a major practical contribution to the field of civil engineering.
- The comparison in terms of absolute displacement suggests that the GFRP frames have good overall deformability (independent of the specific value of damping considered). All three GFRP frames show a homogeneous displacement distribution along the height of the structure. This behaviour is halfway between the ones of the other two traditional materials.
- The time histories of Von Mises equivalent stress in critical nodes reveals that the frames in GFRP show a more regular behaviour than the other materials. In node 5, compared to steel, GFRP presents a mean stress value that is almost three times lower. Moreover, the RC frame has peaks six times higher than its mean value, while the GFRP peaks are only three times larger than its mean value at maximum.
- The composite material shows inter-storey drift ratios that, on some floors, are larger than those of steel. In a generic building, this could cause damage to the infills, floors, or other non-structural elements.
- The GFRP frames have a less uniform stress distribution in the frames than traditional materials. This suggests that optimising beam and column cross-sections could lead to more efficient structural designs.

All outcomes indicate a good performance of the GFRP frame in this comparative analysis under seismic conditions, with a general behaviour that is intermediate between steel and reinforced concrete.

It is important to recall that the aim of this study is limited to the linear response of the three materials under investigation. Material non-linearities, damage-induced non-linearities, failure mechanisms, or other more advanced aspects have not been considered as, without experimental data to properly calibrate them, they would introduce uncertainties in the FE models. Hence, future studies with non-linear elastic/elastoplastic analyses will be necessary to evaluate the seismic response of the GFRP frames and include all factors affecting the material behaviour, parametric analyses, and different seismic loads with different magnitudes. However, even at this first stage, the results reported here highlight the potential value of GFRP as an alternative construction material in seismic areas.

## Figures and Tables

**Table 1:** Kobe earthquake (1995) characteristics.

Earthquake (Epicentre and Date)	Station Name	Component	Magnitude	Distance {km}	PGA {cm/s^2^}
Kobe 1995	KOBE-JMA	90° (East–West)	6.9	0.6	599

**Table 2:** Characteristics of GFRP structural elements.

Element	Moment of Inertia J {cm^4^}	Length L {cm}	Cross-Section Area A {cm^2^}	Weight W {kg}
I-beam, $J_{max}$	209.22	300	14.72	8.65
Q-beam	492.00	240	36.00	14.87

**Table 3:** Mechanical characteristics of GFRP beams.

Longitudinal Elastic Modulus $E_{z}$ {MPa}	Transversal Elastic Modulus $E_{x}=E_{y}$ {MPa}	Transversal Shear Modulus $G_{xy}$ {MPa}	Shear Modulus $G_{zx}=G_{zy}$ {MPa}	Longitudinal Poisson’s Ratio $\nu_{xy}$ {-}	Transversal Poisson’s Ratio $\nu_{zx}=\nu_{zy}$ {-}	Density ϒ {kg/m^3^}
23,000	8500	3455	3000	0.23	0.09	1700

**Table 4:** Structural beams’ pulsations and Rayleigh coefficients.

Element	Pulsation ω {Hz}		Rayleigh Coefficients {-}	
	1st Mode	2nd Mode	α	β
I-beam, $J_{max}$	148.71	569.45	6.1081	7.2129 × 10^−5^
Q-beam	244.95	920.61	10.5249	4.6673 × 10^−5^

**Table 5:** Beam and column profiles for the steel frame.

Columns				Beams	
**T1**		**T2**		**T1**	**T2**
Lateral	Central	Lateral	Central		
HEB200	HEB240	HEB160	HEB180	IPE360	IPE360

**Table 6:** Steel and reinforced concrete mechanical properties.

Material	Elastic Modulus E {MPa}	Poisson’s Ratio ν {-}	Density ϒ {kg/m^3^}
Steel	210,000	0.3	7850
Concrete	30,000	0.2	2500

**Table 7:** Reinforced concrete beam and column equivalent cross-sections.

Steel Profiles	$J_{c}$ Required {cm^4^}	Reinforced Concrete Equivalent Cross-Section {cm}	J Provided {cm^4^}
HEB 160	45,052.80	SQUARE 30 × 30	67,500.00
HEB 180	69,058.67	SQUARE 30 × 30	67,500.00
HEB 200	101,485.33	SQUARE 35 × 35	125,052.08
HEB 240	198,765.33	SQUARE 40 × 40	213,333.33
IPE 360	108,466.67	RECTANGULAR 30 × 40	118,159.74

**Table 8:** RC Structural elements’ reinforcement.

Cross-Section	Area A {cm^2^}	$A_{s,min}$ {cm^2^}	$A_{s}$ {cm^2^}	
SQUARE 30 × 30	900.00	9.00	2+2 ϕ20	12.57
SQUARE 35 × 35	1225.00	12.30	2+2 ϕ20	12.57
SQUARE 40 × 40	1600.00	16.00	3+3 ϕ20	18.85
RECTANGULAR 30 × 40	1200.00	-	4+2 ϕ20	18.85

**Table 9:** Full-concrete beam and column equivalent cross sections.

RC Section	Reinforcement	Concrete Equivalent Section {cm}
COLUMN 30 × 30	2+2 ϕ20	SQUARE 33 × 33
COLUMN 35 × 35	2+2 ϕ20	SQUARE 38 × 38
COLUMN 40 × 40	3+3 ϕ20	SQUARE 44 × 44
BEAM 30 × 40	4+2 ϕ20	RECTANGULAR 30 × 37

**Table 10:** GFRP beam and column profiles.

Steel Profiles	b {cm}	h {cm}	$t_{f}$ {cm}	$t_{w}$ {cm}	$J_{min,GFRP}$ Required {cm^4^}	GFRP Equivalent Section	J Provided {cm^4^}
HEB 160	23.0	23.0	1.2	1.2	7732.17	BOX 230 × 230 × 12	8313.30
HEB 180	25.0	25.0	1.5	1.5	11,852.17	BOX 250 × 250 × 15	13,030.75
HEB 200	28.0	28.0	1.5	1.5	17,417.39	BOX 280 × 280 × 15	18,669.25
HEB 240	33.0	33.0	1.7	1.7	34,113.04	BOX 330 × 330 × 17	34,855.39
IPE360	46.0	48.0	2.5	1.7	141,478.26	BOX 480 × 460 × 17 × 25	141,686.15

**Table 11:** Damping ratios for each material.

	Reinforced Concrete	Steel	GFRP (Box Section)
Damping Ratio ξ	5% ^(a)^	2% ^(b)^	2.652% ^(c)^, 2.720% ^(d)^, and 2.788% ^(e)^

^(a)^ Papageorgiou and Gantes [18]; ^(b)^ Papageorgiou and Gantes [18]; ^(c)^ Boscato and Russo [6,14], Russo [7]; and ^(d,e)^ Parametric Analysis.

**Table 12:** Modes of vibration of all five frames: frequencies and corresponding periods.

	Steel		GFRP (c), (d), (f)		RC	
Mode	Frequency {Hz}	Period T {s}	Frequency {Hz}	Period T {s}	Frequency {Hz}	Period T {s}
1	2.94	0.34	2.04	0.49	1.93	0.52
2	7.89	0.13	5.41	0.18	5.70	0.18
3	14.40	0.07	9.60	0.10	10.73	0.09
4	20.46	0.05	13.34	0.07	16.40	0.06
5	25.65	0.04	15.79	0.06	21.75	0.05

**Table 13:** Maximum shear force at the frame base.

Material	Max Base Shear {kN}
Reinforced Concrete (a)	270.7
Steel (b)	52.4
GFRP (c) ξ = 2.652%	**31.2**
GFRP (d) ξ = 2.720%	31.3
GFRP (e) ξ = 2.788%	33.0

## Data Availability

The data supporting this research are available from the authors upon reasonable request.
